# Supplementary material for: α-Phenylalanyl tRNA synthetase competes with Notch signaling through its N-terminal domain
Source: PLoS Genet. 2022 Apr 29;18(4):e1010185. doi: 10.1371/journal.pgen.1010185 (PMC9094542; doi:10.1371/journal.pgen.1010185)
Supplement: S1 Fig — The kinetics of the Notch knockdown resembled partially the one of PheRS(Cys) expression (esgts / UAS-α-PheRS(Cys)). Animals were mated at 18°C, and adults of the required genotypes were collected and shifted to 29°C to inactivate Gal80ts. Adult midguts were dissected from female flies after the indicated induction times. (PDF) [file pgen.1010185.s001.pdf]

Figure S1

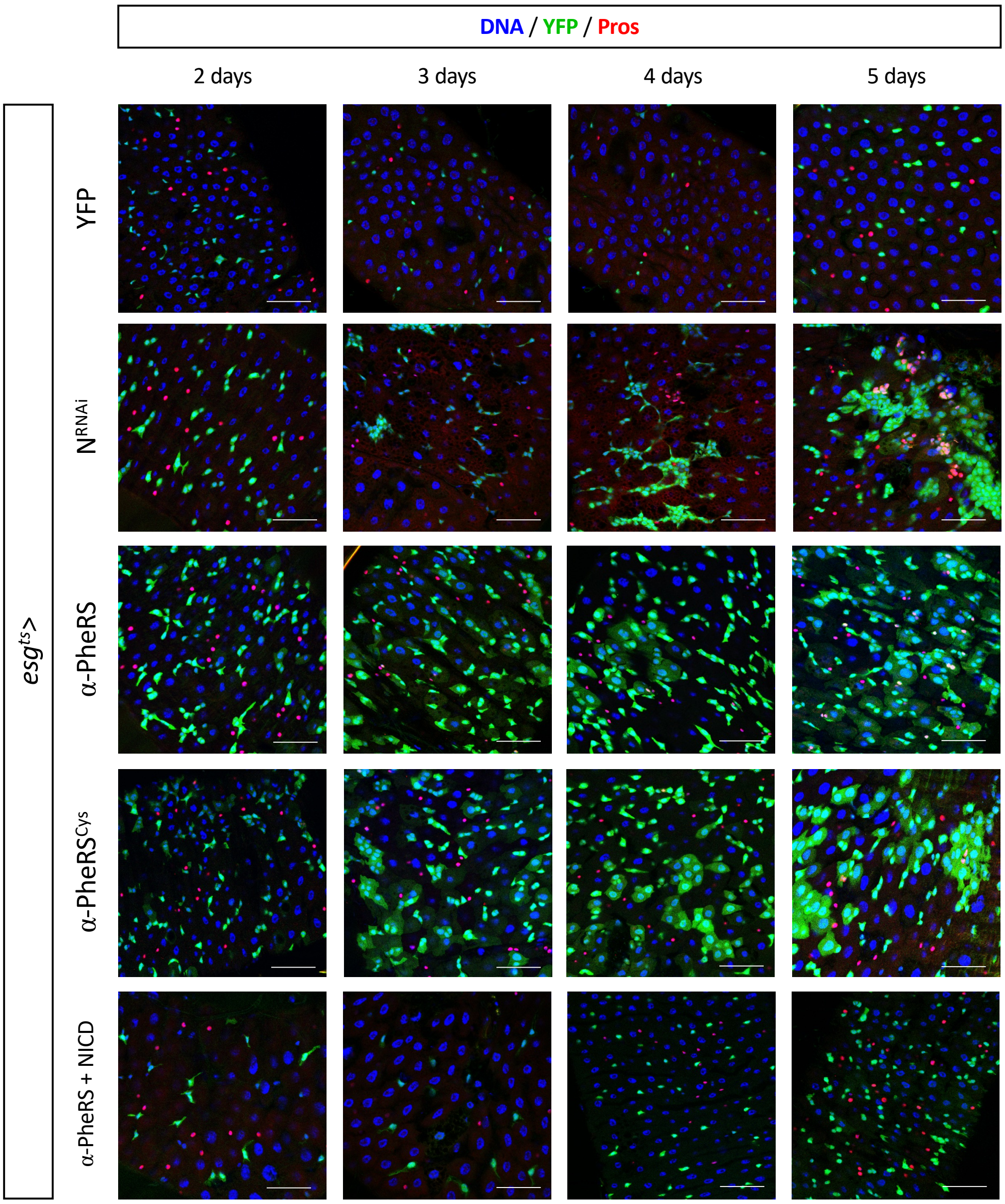

Figure S1: *α-PheRS* or *α-PheRS^{Cys}* were overexpressed using the *esg-Gal4,UAS-2XEYFP;tub-Gal80^{ts}* (=esg^{ts}) system that allowed us to control the expression time to study the kinetics of the appearance of the phenotypes. The kinetics of the *Notch* knockdown resembled partially the one of *PheRS$^{(Cys)}$* expression (esg^{ts} / *UAS-α-PheRS$^{(Cys)}$)*. Animals were mated at 18°C, and adults of the required genotypes were collected and shifted to 29°C to inactivate Gal80^{ts}. Adult midguts were dissected from female flies after the indicated induction times.
