# Supplementary material for: α-Phenylalanyl tRNA synthetase competes with Notch signaling through its N-terminal domain
Source: PLoS Genet. 2022 Apr 29;18(4):e1010185. doi: 10.1371/journal.pgen.1010185 (PMC9094542; doi:10.1371/journal.pgen.1010185)
Supplement: S2 Fig — The en-Gal4,UAS-EGFP;tub-Gal80ts (ents) system was used to drive transgene expression in the posterior compartment of developing wing discs (ents/UAS-Myc::α-S or Myc::α-SW112A) and Hoechst staining to label nuclei. Animals were initially kept at 18°C for 3 days and then shifted to 29°C to inactivate Gal80ts until adult flies hatched, enabling expression of Myc::α-S or Myc::α-SW112A. (PDF) [file pgen.1010185.s002.pdf]

Figure S2

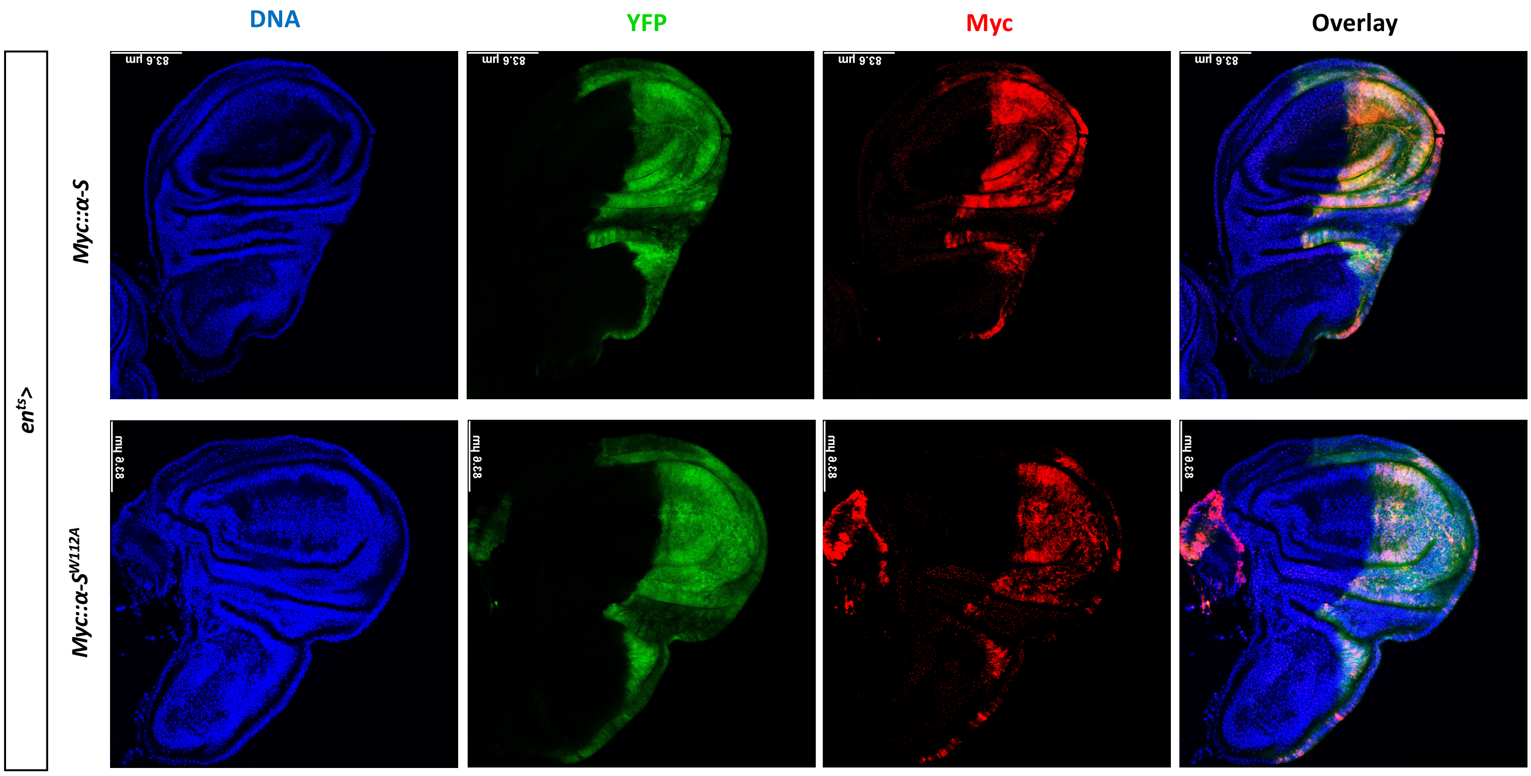

Figure S2: Staining wing discs for Myc::α-S showed the stable expression of both truncated alleles *Myc::α-S* and *Myc::α-S^{W112A}*. The *en-Gal4,UAS-EGFP;tub-Gal80^{ts}* (en^{ts}) system was used to drive transgene expression in the posterior compartment of developing wing discs *(*en^{ts}*/UAS-Myc::α-S* or *Myc::α-S^{W112A}*) and Hoechst staining to label nuclei. Animals were initially kept at 18°C for 3 days and then shifted to 29°C to inactivate Gal80^{ts} until adult flies hatched, enabling expression of Myc::α-S or Myc::α-S^{W112A}.
