# Supplementary material for: α-Phenylalanyl tRNA synthetase competes with Notch signaling through its N-terminal domain
Source: PLoS Genet. 2022 Apr 29;18(4):e1010185. doi: 10.1371/journal.pgen.1010185 (PMC9094542; doi:10.1371/journal.pgen.1010185)
Supplement: S3 Fig — The tryptic peptide fragments of the 25kDa band were subsequently analyzed by mass spectrometry (MS). The MS data analysis revealed the peptide coverage of the 25KDa isoform according to the score of Peptide Spectrum Matches (PSM). The 25 KDa isoform contains the peptides of the N-terminal 28% of the full-length α-PheRS. (PDF) [file pgen.1010185.s003.pdf]

Figure S3

MS data of 25 kDa isoform

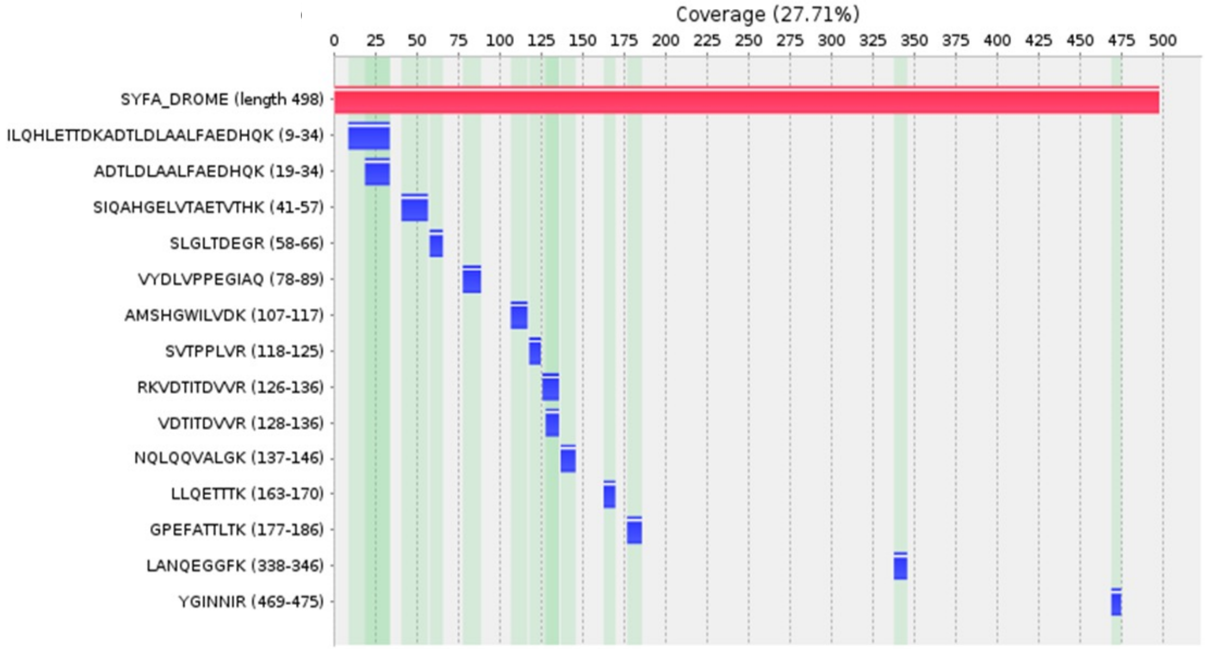

Figure S3: The Myc-tagged isoforms from whole larvae were purified by immunoprecipitation and gel purification. The tryptic peptide fragments of the 25kDa band were subsequently analyzed by mass spectrometry (MS). The MS data analysis revealed the peptide coverage of the 25KDa isoform according to the score of Peptide Spectrum Matches (PSM). The 25 KDa isoform contains the peptides of the N-terminal 28% of the full-length α-PheRS.
