# Supplementary material for: α-Phenylalanyl tRNA synthetase competes with Notch signaling through its N-terminal domain
Source: PLoS Genet. 2022 Apr 29;18(4):e1010185. doi: 10.1371/journal.pgen.1010185 (PMC9094542; doi:10.1371/journal.pgen.1010185)
Supplement: S4 Fig — An ectopic vein branches from the connecting vein between the L4 and L5 vein (arrowhead in B-F). D) Knockdown of Notch shows this phenotype and the classical notched phenotype in the distal region of L3, L4, and near the L5 vein. The en-Gal4,tub-Gal80ts (ents) system was used to drive transgene expression in the posterior compartment of developing wing discs (ents/UAS-α-PheRS(Cys)). Animals were initially kept at 18°C for 3 days and then shifted to 29°C to inactivate Gal80ts until adult flies hatched, enabling expression of α-PheRS, α-PheRSCys, α-S, or NRNAi. (PDF) [file pgen.1010185.s004.pdf]

Figure S4

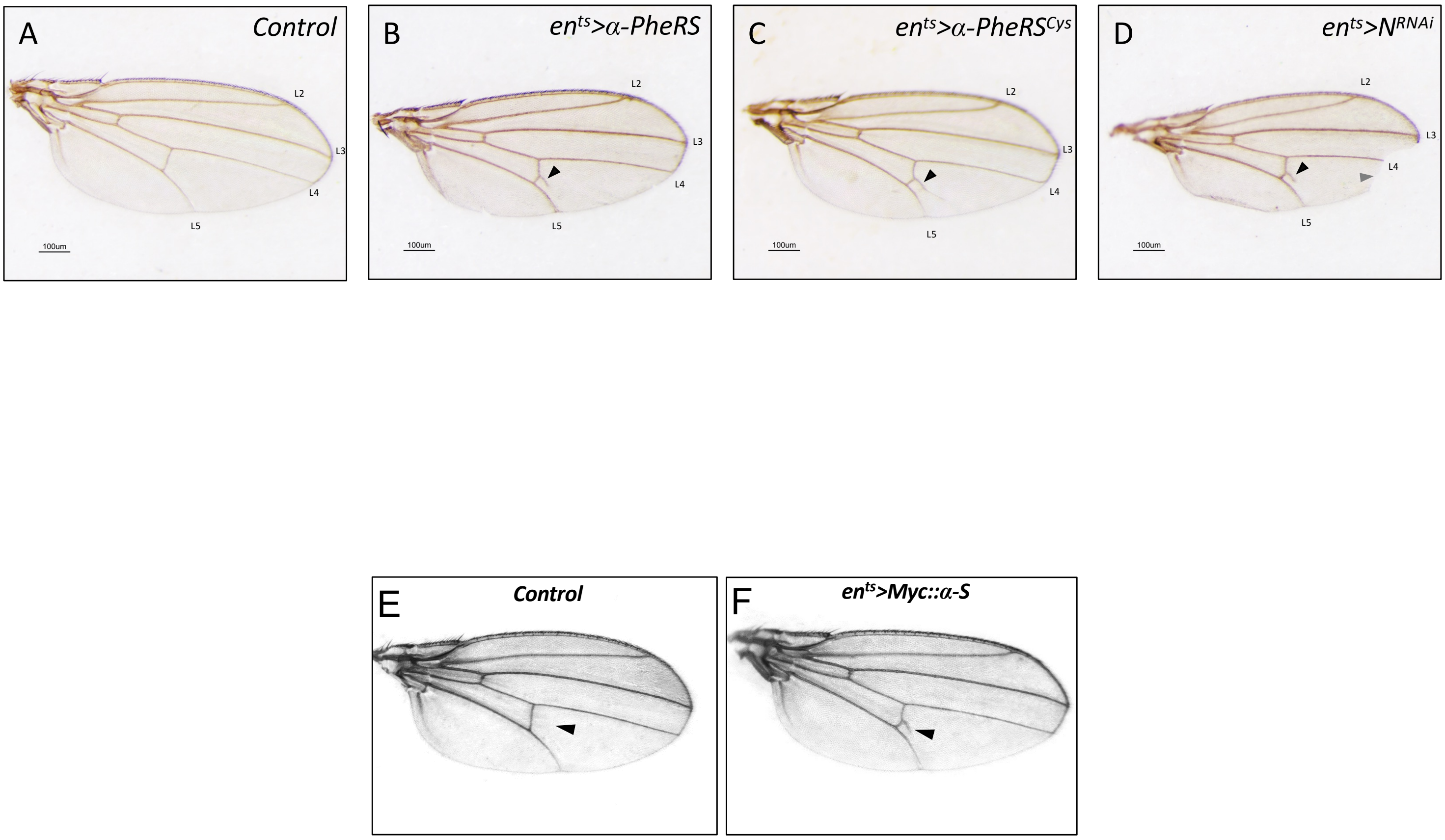

Figure S4: The ectopic wing venation phenotype resulting from elevated levels of α-PheRS$^{(Cys)}$ and α-S is similar to the phenotype of *N*^{RNAi} treatment. An ectopic vein branches from the connecting vein between the L4 and L5 vein (arrowhead in B-F). D) Knockdown of *Notch* shows this phenotype and the classical notched phenotype in the distal region of L3, L4, and near the L5 vein. The *en-Gal4,tub-Gal80*^{ts} (en^{ts}) system was used to drive transgene expression in the posterior compartment of developing wing discs *(*en^{ts}*/UAS-α-PheRS*$^{(Cys)}$*).* Animals were initially kept at 18°C for 3 days and then shifted to 29°C to inactivate Gal80^{ts} until adult flies hatched, enabling expression of α-PheRS, α-PheRS^{Cys}, α-S, or *N*^{RNAi}.
