# Supplementary material for: α-Phenylalanyl tRNA synthetase competes with Notch signaling through its N-terminal domain
Source: PLoS Genet. 2022 Apr 29;18(4):e1010185. doi: 10.1371/journal.pgen.1010185 (PMC9094542; doi:10.1371/journal.pgen.1010185)
Supplement: Attachment — Submitted filename: Responses to Reviewers, Ho et al.pdf [file pgen.1010185.s006.pdf]

Reviewer #1: Ho et al. aims to determine whether and how alpha-Phenylalanyl tRNA synthetase (α-PheRS) promotes cellular growth and proliferation in tissue progenitors and how it may contribute to tumour formation.

Ho et al. suggest that α-PheRS blocks Notch signalling by competing with the Notch intracellular domain (NICD) for factors that promote downstream Notch target expression. The authors provided a series of experiments in a variety of tissues (larval and adult midgut, larval brain, larval wing imaginal discs and adult wings) to show that α-PheRS overexpression resembles the loss of Notch in larval and adult midgut progenitors, larval neuroblasts and wing imaginal disc cells. These effects included progenitor cell and enteroendocrine cell (EE) expansion in the adult midgut, the loss of type 1 and 2 neuroblasts in the larval midgut, and ectopic vein formation in the adult wing. Furthermore, the authors showed similar effects by overexpressing a likely aminoacylation-dead α-PheRS (α-PheRSCys), suggesting that the effects are not translation-dependent. They also showed that α-PheRS and α-PheRSCys overexpression can inhibit the Notch-responsive reporter, NRE-GFP, in the adult midgut and larval wing disc. Lastly, the authors suggest that α-PheRS competes to bind factors that interact with the NICD, such as Su(H). They provide experiments to support this hypothesis by attempting to rescue the effects of α-PheRS overexpression by NCID expression. Additionally, they isolated an N-terminal region of α-PheRS that may compete with NICD. To do this, the authors generated a truncated N-terminal-only α-PheRS (α-S), which when overexpressed can produce the same effects in adult midguts and larval and adult wings as the full-length version. Using Western analysis, the authors showed that α-PheRS is normally found in larvae in a variety of forms (55, 40 and 25 KDa) and that different forms can be found in different larval tissues. Using mass spectroscopy, the authors identified regions in the smaller variant (25 KDa) that contains overlapping regions with α-S. Using protein sequence analysis, the authors further identified a conserved tryptophan in α-PheRS found in RAM domains of NICD that may aid in binding to other factors. Finally, the authors showed that the effects of α-PheRS overexpression in the adult midgut are lost by the expression of the W112A mutant version.

While the authors did show effects of α-PheRS overexpression and the importance of its N-terminal region in mediating these effects, the evidence that the authors provided for its role in antagonising Notch is not convincing. This is mainly because the effects of alpha-PheRS overexpression often do not or only somewhat resemble the loss of Notch signalling in these tissues. Since there in no biochemical evidence provided that α-PheRS competes for factors that interact with NICD nor that the W112A mutation abrogates its binding to these factors, strong evidence is needed to claim that α-PheRS overexpression affects Notch signalling. Based on their data in the larval and adult midgut and their previous work (Ho et al., 2020), it does seem that α-PheRS plays a role in proliferation in certain contexts.

Major concerns:

1. The effects of α-PheRS overexpression often do not or only partially resemble the loss of Notch signalling.

a) Adult midgut: the authors claim that the α-PheRS or α-PheRSCys overexpression resembles the loss of Notch signalling in adult midgut progenitors by showing that α-PheRS or α-PheRSCys overexpression results in the expansion of escargot (esg)+ cells and Prospero (Pros)+ EEs (Fig. 1E, 2). While the quantification does show a mild increase in EE cells upon α-PheRS overexpression, the images in Fig. 1 do not show an increase in EE cell clusters characteristic of the loss of Notch in adult midgut progenitors (See Ohlstein and Spradling, 2007, Patel et al., 2015). Loss of Notch in adult midguts results in the expansion of cells that resemble ISCs/progenitors and express high levels of ISC or progenitor (Delta, E-cadherin) and EE (Pros, Sc) markers (Maeda et al., 2008, Patel et al, 2015, Chen et al., 2018).
It's possible that the esg+ and EE cell number increases due to accelerated ISC proliferation and tissue hyperplasia. In support, the α-PheRS overexpression results in a mild increase in mitotic cells and hyperplasia in the larval gut (Fig. 3F-G). The authors don't, however, specifically test (e.g., by clonal analysis) whether PheRS overexpression affects cell number or cell size (growth) in the adult midgut.

We agree that we had focused more on the overlapping phenotypes than on the differences between the phenotypes. We fully agree with the observation of this reviewer that there is an increased number of esg+ cells and EEs. But it is also true that there are differences between the phenotypes, and these can be expected. For one thing, because α-PheRS might not only function in the N signaling pathway (its proliferative effect on ISCs that we have described in our previous publication (Ho et al, 2021) seems to be – in part – not through *N*). Similarly, both Notch and Su(H) do not only function through the Notch - Su(H) pathway (e.g., Sanders et al., 2009; Johnson and Macdonald 2011), they interact with other signaling pathways and transcription factors. For example, as opposed to the knock down of *N,* in the case of elevated α-PheRS we expect that there is still plenty of N present in the cell and on the plasma membrane. This N could still perform the Su(H)-independent function(s). Directly or through feedback loops, the remaining N protein might be responsible for the phenotypic differences. Similarly, in the absence of N, α-S might still perform an activity outside of Notch signaling. We have now incorporated these points in the new manuscript.

We had previously attempted to perform MARCM clone analyses in guts. However, in our hands, the cell fate changes resulting in different size cells make this tissue too difficult for such an analysis. For this reason, we had resorted to a MARCM analysis in follicle cells where this complication does not exist (Ho et al, 2021). In the Ho paper we described a clear increase in cell numbers when overexpressing α-PheRS. At the single cell level, the higher α-PheRS levels did not affect cell size, but the total clone size and the cell numbers increased with the higher α-PheRS levels.

Furthermore, in Fig.2E and H, it is unusual that the midgut is devoid of EEs after the NCID expression because the midgut takes several (3 or more) weeks to turnover (Jiang et al., 2009, Antonello et al., 2015). NICD expression in progenitors results in their rapid differentiation into ECs without affecting existing Pros+ EEs.

We agree that this is an unusual phenotype, but previous publications also suggested that this happens (Micchelli and Perrimon, 2006; Zhai, Boquete, and Lemaitre, 2017). This could actually mean that NICD also accelerates turn-over of the gut tissue. Or it could mean that NICD stopped the expression of the Prospero marker. It would be beyond the scope of this manuscript to solve this issue because for the interaction between N and PheRS we needed to focus primarily on the effect on the ISCs/EBs and ECs. The EE differences affect the frequency of the other cells much less. To deal with this issue we have therefore added the sentence “Whether and why NICD expression caused EEs to disappear in this short period of time or only to lose expression of Prospero is not known to our knowledge.”

The NRE-GFP levels in the adult midgut were measured using a Western blot. It would be more convincing to see NRE-GFP levels in the adult gut as shown for the larval wing disc.

We are not sure whether we understand this point correctly. If we understand it as a recommendation to analyze the NRE-GFP in the adult gut by immunostaining, we did not do this because, as opposed to the wing, we do not have an endogenous control compartment that serves as a negative control.

b) Larval midgut: It is not clear at which larval stage the midguts in Fig. 3A-D were examined (Jiang and Edgar, 2009). Although there seems to be a similar increase in AMP numbers after α-PheRS overexpression or N loss in AMPs, it is not clear that the effects of α-PheRS overexpression resemble Notch. For example, does the increase in AMP number after α-

PheRS overexpression result in an increase in EE precursors or peripheral cell loss in pupal midguts as described in Takashima et al., 2001 and Mathur et al., 2010?

We tested late third instar larvae (wandering larvae).

These experiments serve to show the phenotypic similarities between the effects of elevated α-PheRS levels at the two stages. Also in this case, the overexpression of the aminoacylation-dead version of α-PheRS (α-PheRS^{Cys}) leads to a highly similar phenotype as Notch knockdown. We observe hyperplasia and/or dysplasia at the same or even at a more severe level. We also add the result of increased EE proportion when overexpressing α-PheRS (Fig 3M).

c) Adult wing: the authors showed that α-PheRS overexpression does not result in a notched-wing; however, it causes ectopic vein formation. This suggests perhaps a mild effect on Notch signalling, but the ectopic vein formation could also be from effects on other pathways.

We now moved this part to the supplementary material. We now bring up different explanations for this phenotype in the discussion and mention that the experiment does not allow us to discriminate between them.

d) Larval wing disc: the authors showed that α-PheRS overexpression in the posterior compartment results in a complete loss in NRE-GFP, suggesting a strong loss of Notch signalling. However, the α-PheRS overexpression does not cause a massive overgrowth (or duplication?) in the disc, which can be observed in the loss of Notch disc in Fig. 5F. This disc abnormality or overgrowth (or the lack of in discs overexpressing α-PheRS) are too not described in the text.

There appear to be different effects on the anterior compartment between the α-PheRS^{Cys} and N^{RNAi} treatments in the posterior compartment. Because these effects are in the untreated control compartment, they would appear to be more indirect than the effect on the NRE-eGFP reporter and have therefore not been followed up. We wish to point out that the purpose of this experiment was to visualize the effect of α-PheRS($^{Cys)}$ on N-signaling.

The larval and adult wing data provided are incongruent. The effects of α-PheRS on the adult wing (ectopic vein formation) and larval wing (no disc overgrowth) suggest a mild effect on Notch. In contrast, α-PheRS overexpression in discs causes the loss of NRE, suggesting a strong effect on Notch. The authors need to reconcile these differences.

We favor another interpretation and do not see the adult wing phenotype as a mild effect, but more likely being caused by different requirements/pathways. For instance, the additional pH3-positive cells in the posterior compartment where there is no Notch might point to a compensatory mechanism that produces the cells required to reach proper organ size. We cover these issues now in the Discussion.

Perhaps a further analysis of Dl expression in establishing D/V Notch signalling or an analysis of Notch targets Wg or cut will help clarify this more.

Thank you for your suggestion. In the context of pointing out the Su(H)-independent function of Notch, we are now also pointing out the reported effect on Armadillo. The main point of this experiment was to show convincingly the effect on Notch signaling through Su(H). In our

mind, additional, interesting questions that these results bring up, will have to be addressed later.

e) Type 1 and type 2 neuroblasts: In contrast to what the authors claim, Notch is not involved in type 1 neuroblast maintenance, but is involved in type 2 neuroblast maintenance (Haenfler et al., 2012, Xiao et al., 2012, Li et al., 2016). It is not clear how type 2 neuroblasts are lost from the central brain upon α-PheRS overexpression. Are they lost due to cell death or by differentiation or transformation to type 1? The authors also do not show any further molecular changes that occur due to loss of Notch in type 2 neuroblasts (e.g., erm expression, Li et al., 2016). Although the central brain area is shown, the effect of the loss of Notch and the effect of α-PheRSCys and NICD on type 1 neuroblasts should be shown in Fig. 4.

With this experiment we only wanted to test whether α-PheRS indeed acts on Notch signaling or whether it affects primarily proliferation and not Notch signaling. This system allowed us to do this because in NBs, N signaling has the opposite effect on proliferation vs differentiation. Figuring out the details of this pathway could easily be an independent project.

We added the images for loss of Notch and the effect of α-PheRS^{Cys} and NICD on type 1 neuroblasts as requested.

2) NICD experiments: the authors used NICD to rescue the effects of α-PheRS overexpression. In the adult fly midgut, the overexpression of NICD results in rapid progenitor differentiation into ECs (Ohlstein and Spradling, 2007; Korzelius et al., 2014). Thus, it is not surprising that its expression suppresses esg+ cell expansion or reduces the number of abnormal, partially-differentiated ECs upon α-PheRS overexpression.

Yes, we agree with this view. This result on its own could lead to different models. But it is consistent with our model and thus an important piece of the puzzle.

3) W112A mutant: The authors showed that α-PheRS W112A mutant overexpression is unable to have the same effects of α-PheRS overexpression on tissue homeostasis, suggesting that this W is critical for ability of α-PheRS to compete with NICD. However, it is not clear if the W112A mutant form is stably expressed. It is also not clear whether there is high enough homology between α-PheRS and Notch RAM domains to be certain that a domain similar to RAM exists within α-PheRS.

We now provide the images from staining wing disc to prove that the mutant W112A is stably expressed and able to translocate to the nucleus just as the wild-type version.

Minor concerns:

1. The authors showed an enrichment of α-PheRS in what are likely larval and adult midgut progenitors (Figs. 1A,C). Using a marker (e.g., esg) to mark the progenitors will solidify the claim that PheRS is enriched in progenitors. Is α-PheRS enriched in other progenitors?

We now provide images (Figure 1E-F””) that show the staining for α-PheRS expressed from the endogenous *α-PheRS* promoter in progenitor cells (esg^{ts}>YFP) and in EEs (Pros+ cells).

We have not looked yet for accumulation in other stem cells. Particularly interesting would be the ones in the ovary. It has been on the agenda, but…

2. The experiments in Fig. 1E should be part of Fig. 2. Fig. 1 does nicely show a time series of the development of the effects of α-PheRS overexpression. Similar data can be found in Fig. 2 A-F for 5 days. As they showed similar effects at 5 days, perhaps the data in Fig. 1E could be placed into supplemental data or selectively combined with Fig. 2. The data also in 3H is relevant to Fig. 2 and not Fig. 3, which focuses on the effects of α-PheRS on larval progenitors.

We moved the Fig1E into the Supplementary data (now as Suppl. Fig S1). Fig 3H is now Fig 2J.

3. The authors suggest that they are counting "differentiating EBs." From the text, it seems that they mean ECs that continue to express esg+ cells. Perhaps a marker for ECs (e.g., Pdm1 or Myo1A) could be used together with esg to show that these abnormal ECs still express progenitor markers. This is often observed with midgut hyperplasia or dysplasia in aging midguts (Biteau and Jasper, 2008).

We would have liked to try these experiemnts, too. We had contacted the lab that produced the Pdm1 antibody two times to ask for the antibody. However, they already used up all their anti-Pdm1 supply. We are aware of the Myo1A-Gal4 (NP1-GAL4) fly lines that can be used as markers, but our use of the Gal4 system for increasing α-PheRS levels in ISCs prevents us from using it (it would also be difficult to get all the genetic elements together).

4. The y-axis in Fig. 2H would be clearer if labelled %EE/total cells per region. Similarly, it should be edited for Fig. 2I. Total cells should include all cells in this case and not only ECs. EEs make up roughly 10% of all midgut cells (Ohlstein and Spradling, 2007).

We changed the labels of the y-axis as recommended. The "total cells" includes here all cell types, not only ECs.

5. In Fig. 4B, the y-axis could be clearer. Perhaps "ratio central brain/optic lobe size".

We corrected the y-axis labels as recommended (4B has become 4G).

6. In the figures, insets from images (e.g., Fig. 3 A'-D') or images of different specimens (Fig. 4A) should not be labelled with ' or ''. Using ' suggests that each image are different channels of the first image presented. These should be labelled as separate panels as in Fig. 1A-D.

We corrected the labels as recommended.

7. Scale bars are missing from panels C-H. Scale bars can be found in some panels but are missing from many others.

We corrected the scale bars as recommended.

8. The titles of subsections and figure legends do not accurately describe the findings and can be more accurate.

We rephrased the legends as recommended.

9. In Fig. 4, the authors should make it clear that GFP is being shown in each panel.

We corrected the labels as recommended.

10. esg is recessive, and thus should be written esg, not Esg. See Fig. 3.

We corrected the labels as recommended.

11. A discussion of which cancers show upregulated α-PheRS and whether Notch signalling is tumour suppressive in these tissues will help express the significance of the work better.

We mentioned some details in the discussion section.

12. The manuscript needs careful editing for clarity and accuracy.

Thank you for comments. We carefully checked and rephrased the manuscript.

Reviewer 2:

The authors identify the alpha subunit of the cytoplasmic Phenylalanyl tRNA synthetase (α-PheRS) as a novel antagonist of the Notch signaling pathway in drosophila.

Using defined fly strains, to express or knock-down, the components/targets in a tissue and/or temperature dependent manner, they show convincingly repression of Notch phenotypes and downregulation of a Notch responsive reporter by α-PheRS in three different settings: midgut, neuroblasts (NBs) and wing formation.

They also show that the N-terminal part of the protein (alpha-S) is sufficient for repression. In addition, a tryptophan residue within alpha-S seems to be essential for repression of the Notch signaling pathway. Mechanistically, they propose a model of action (Notch repression) in which α-PheRS (alpha-S) competes for NICD/Su(H) interaction by binding to the BTD of Su(H) as the RAM domain of NICD does. This mechanism was shown to be realized by RBPJ-corepressor action in vertebrates.

The MS is well written and the experiments are sound and conclusive. I have to congratulate the authors for this peace of work. If true, they discovered a novel mechanism for Notch modulation (here repression) in the fly, not known so far.

However, showing the displacement mechanism to repress Notch needs to be improved. Therefore, I think some points have to be clarified by experiments and/or discussion prior to publication:

1. Although the authors mention a compensation of alpha-PheRS(W112A) for the wt form regarding the Phe-t-RNA loading function, expression of alpha-S(W112A) and alpha-PheRS(W112A) should be shown by WB (anti-Myc).

We now provide the staining of posterior compartments of wing discs (with the anterior compartment as internal control) to show that alpha-S^{W112A} is as stably expressed as alpha-S (Fig S2) and also found in the nucleus (Fig 7I-L). Expression levels and subcellular distribution are therefore not affected by the mutation.

2. Is alpha-PheRS (alphaS) located in the nucleus and are the mutated forms still located in the nucleus? (microscopy using higher magnification should be performed).

alpha-PheRS is mostly cytoplasmic. alpha-S^{W112A} is as stably expressed as alpha-S (Fig S2) and both are also found in the nucleus (Fig 7I-L). Expression levels and subcellular distribution are therefore not affected by the mutation.

3. To strengthen the proposed model of action of alpha-PheRS repressing Notch signaling (which is weak so far), Co-IPs with alpha-PheRS(wt), alpha-S(wt), together with Su(H) are necessary. alpha-PheRS (W112A) and/or alpha-S(W112A) should be used as control.

If the N-terminal part of alpha-S (especially the residues around the tryptophan (HGWIL) is conserved also in vertebrates (alignment as additional figure), these experiments could also be performed using vertebrate proteins.

We agree that this is an important experiment that would give the direct evidence for the alpha-PheRS interaction with Notch signaling. In the limited time we had and under the present circumstances it was not possible to show this interaction. We tried to do this in Drosophila and not in cells. However, because getting enough gut tissue for this experiment did not seem possible, we resorted to embryos. And in these, we did not see the interaction. At this point it is unclear whether we need to improve the protocol. If, for instance, DNA binding through the DBD is needed, we would need to isolate soluble DNA/Su(H)/a-S complexes. We also had problems with the only available Su(H) antibody (from Santa Cruz) that did not perform well in WBs. And, additionally, we might have to use different stages, organs or maybe even expression in tissue culture cells. Because it was not possible to obtain the expected results in the meantime, we now phrase the results and the discussion of the possible competition for Su(H) binding only as a possibility.
Nevertheless, we would like to point out that the NRE-EGFP with its 2 paired Su(H) binding-sites is repressed by α-S (and not by the mutant). This tells us that α-S has to repress Notch signaling at or before the transcription of this reporter.

4. Does the proposed displacement mechanism occur on Notch target genes?

A ChIP analysis would be helpful but might also be a difficult task in drosophila tissues.

This would be a great experiment. However, it goes beyond our capacity to do this during the revision.

Reviewer #3: In this manuscript Ho et al. investigated the role of the alpha subunit of the cytoplasmatic Phenylalanyl tRNA synthetase (α-PheRS) as a novel general repressor of Notch signalling. They showed an inhibitory effect on Notch signalling in different larval and adult Drosophila tissues by similarities of α-PheRS overexpression to Notch-RNAi phenotypes that were rescued by simultaneous expression of the Notch intracellular domain. Besides, the authors proved that protein levels of a Notch responsive element reporter are reduced upon overexpression of α-PheRS. Thereby expression of an α-PheRS variant (α-S) encoding for the N-terminal 200 amino acids and lacking the catalytic domain was sufficient to induce phenotypes similar to Notch loss of function, indicating that the inhibitory effect on Notch signalling is independent of the catalytic function of α-PheRS. α-S also shows a weak sequence similarity to the Notch intracellular domain which includes a conserved key tryptophan residue which was shown to be essential for α-S to attenuate Notch signalling. Although the paper is well written and overall comprehensive, the figures and data presentation are of poor quality and need revision.

Major comments:
1) Line 145 and following: The authors state that α-PheRS levels are higher in progenitor cells compared to differentiated cells in Drosophila larval and adult midguts. For this statement they compared signal levels of antibody stainings against α-PheRS in diploid cells to polyploid cells, but without using any characterized markers for the different cell types. Enteroendocrine cells and their progenitors are also diploid whereas Enteroblasts already undergo endocycles prior to differentiation and are also polyploid (Edgar et al. 2014). The authors should characterize the different cell types by established markers to clearly distinguish them and discuss with the findings in the Edgar paper and this paper from the Dominguez lab (Antonello et al. 2015). Also, for defining the "differentiating EB" they should use Enterocyte markers like discs large 1 to proof that they are not fully differentiated Enterocytes by negative staining.

This is a good point; we agree that we should add this. We now provide the comparison of α-PheRS, driven by the endogenous *α-PheRS* promoter, and esg^{ts}>*YFP* (to label stem cells and progenitor cells), anti-Prospero staining (for Enteroendocrine cells) as Figure 1E, F. We would have liked to also stain for an EC marker to distinguish between EB and EC. However, the authors who created the Pdm1 antibody (EC marker) told us that they have nothing left. We had also tried the anti-Discs large 1, but in our hands, it labeled all plasma membranes and was therefore not a marker for ECs. Actually, Antonello et al. also stated that it outlines the differentiated cells and not specifically ECs.

2) Line 182: Do the authors have data or in silico predictions which show or predict that α-PheRS directly binds "common downstream targets" of Notch signalling? If not, this speculative statement should better be mentioned in the discussion.

At the end of this part, we only tried to present in one final sentence an idea how this interaction might come about ("They might for instance…" and then two possibilities). We now removed it and only discuss it in the Discussion section.

3) The presence of several RNAi stocks in the material and methods part suggests that also knockdown experiments for the α-PheRS have been performed. This raises several interesting questions:

I. Are they able to accelerate differentiation through RNAi?

N RNAi has been published and reported in many papers to accelerate ISC proliferation and differentiation of EEs, but not ECs. This we also found in our experiments. alpha-PheRS RNAi led to the lethality of the animals when it was expressed in progenitor cells (esg+) of adult guts or ubiquitously. Knock down in the fat body and eye, respectively, was less severe, resulting in smaller pupae and adult eyes, respectively (Ho et al, 2021). Because this was done in our previous paper only, we now removed these entries from the Materials section.

II. Enteroblast specific function downstream of Notch input in adult midguts should be investigated using klumpfuss-Gal4 driver combined with tracing system (Korzelius et al. 2019, Reiff et al. 2019)

Thank you for your suggestion. According to the Klu expression in the intestine, the klumpfuss-Gal4 driver is a very good tool to investigate the role of Notch signaling in the ISC fate decision and also in EB differentiation. We agree that it would be a good choice to deeply investigate the mechanism of α-PheRS on Notch in the intestine. In our study, we chose the NRE-EGFP as another good tool to study effects on Notch signaling, not the least because it has a wider tissue range of expression and therefore allowed us to investigate more general effects of α-PheRS on Notch signaling, not only in the intestine. We were particularly delighted that it showed us very clearly these amazing effects in wing discs (Fig 5,6). Moreover, it turned out that the NRE-EGFP with its 2 paired Su(H) binding-sites was a good choice for further experiments because α-PheRS and α-S repressed its expression and this result became a major argument for our model. In the present version we now emphasize this point better.

III. At least accelerated differentiation needs to be discussed as it was previously observed in e.g. (Korzelius et al. 2014, Korzelius et al. 2019, Reiff et al. 2019, Zipper et al. 2020). At least, these papers should be discussed with the data in the present manuscript.

I agree that this is an interesting point and it should be discussed. We now cover it in the discussion part (second paragraph).

4) Microscope images of the same tissue within one figure should be orientated in the same direction for better comprehensibility. Textboxes and corresponding images should be aligned properly. Diagrams should have precise and coherent axis titles.

We improved this.

5) Figure 3 E – E'' and F: In E'' it looks like there are more PH3+ cells compared to E', but F shows the opposite. The quantifications in F should be normalized to the total cell number. G: is the "intestinal region" somehow defined? 200 cells cannot be the whole gut.

"In E'' it looks like there are more PH3+ cells compared to E', but F shows the opposite".

The two panels 3E' and 3E" (now 3J, 3K) are image examples comparing the effects of elevated α-PheRS and α-PheRS^{Cys} levels in otherwise wild-type guts. There are some cases when α-PheRS^{Cys} had more Ph3+ than α-PheRS^{wt} as summarized in Fig 3L. However, the two sets do not show significant differences (n.s).

"The quantifications in F should be normalized to the total cell number":
We now show a new analysis with the data normalized to the total cell number (Fig 3L,M).

"intestinal region"
It is per frame of microscope objective 63x. This is now mentioned in the legends.

Minor comments:
1) "Drosophila" should be written italic throughout the manuscript

We corrected this as recommended.

2) In the summary (line 39) the authors write about "moderately elevating α-PheRS levels" in adult midguts. What do they mean with "moderately elevating" in an experiment with Gal4/UAS driven overexpression of α-PheRS? Did they perform qPCRs or Western Blots to proof these moderately elevated levels of α-PheRS?

The increase of the protein appears to be rather moderate compared to o/e of many proteins using a strong Gal4 driver. We quantified protein levels and compared between normal level and the overexpression by UAS promotor in immunofluorescence staining of wing discs. The mean value of intensities of the fluorescent signals in the anterior and the posterior compartment were compared after applying the same settings (that did not produce a signal for the secondary antibody) for all image acquisitions. Pixel intensities in the two compartments of the wing discs were then compared. Driving the expression of only a wild-type copy of α-PheRS, the anti α-PheRS antibody produced a mean pixel intensity of 68 ± 9 in the posterior compartment and a mean pixel intensity of 37 ± 7 in the anterior compartment (n=4). The intensity was therefore 1,8 times as high in the posterior. The expression of a missense mutant of α-PheRS alone gave similar results. Posterior mean pixel intensity was 60 ± 13, the anterior one 34 ± 6; posterior levels were thus 1,7 times as high (n=8). Under the assumption that our primary antibody has 0%, 25% and 33%, respectively, cross reactivity, we measured and calculated an increase of the wild-type α-PheRS levels of 80%, 107%, and 120%, respectively. We have no evidence for high cross-reactivity of this antibody but cannot measure it because cells lacking α-PheRS are dead. In general, the expression of α-PheRS or its mutants increased around 1.7~1.8 times compared to normal level. The relatively low increase of the α-PheRS levels is probably also caused because in many differentiated cell types α-PheRS expressed alone is much less stable than the tetrameric $\alpha_2\beta_2$ complex (Lu et la., 2015).

3) In Figure 1 B,D: axis titles of diagrams are too small. E: images can be subtitled with E-E''',F'-F''' and so on, this would make it easier to refer to the different genotypes shown. "esgts>YFP" is hard to read (green letters on grey background), correct throughout all figures.

We moved the Fig 1E with the different genotypes into the Supplementary Data (Figure S1) which has a separate figure legend. The identification of each genotype is now clear.

We also change the background "grey" to "white" to raise the contrast.

4) In Figure 2 H: It should be written what was quantified "percentage of.." like in I.

We corrected the labels as recommended.

5) Figure 4: A and B: Data for insc>NICD is missing.

We provide the data for NICD as recommended.

6) Figure 6: G: y-axis title incomplete, “percentage of..” like in 4)

We corrected the labels as recommended.

7) Figure 7: A: identity and similarity of aligned sequences and a legend should be added.

We added the identity and similarity values in the figure legend.
