# Supplementary material for: α-Phenylalanyl tRNA synthetase competes with Notch signaling through its N-terminal domain
Source: PLoS Genet. 2022 Apr 29;18(4):e1010185. doi: 10.1371/journal.pgen.1010185 (PMC9094542; doi:10.1371/journal.pgen.1010185)
Supplement: Attachment — Submitted filename: Response to Reviewer 1.pdf [file pgen.1010185.s007.pdf]

Reviewer #1: In this revised manuscript, the authors claim that alpha-PheRS blocks Notch signalling by competing with NICD for factors that promote Notch target expression. They claim that the effects of alpha-PheRS overexpression resembles the loss of Notch signalling in larval and adult midgut progenitors, larval neuroblasts and wing imaginal discs. Other than showing alpha-PheRS can block the Notch reporter (NRE-GFP) in larval wing discs, the authors have not convincingly shown that that the other effects they describe in the larval and adult midgut, larval neuroblasts and the adult wing resemble the loss of Notch.

➔ As the reviewer states, we phrase the similarity as "resembles". This we can safely state. We also do not hesitate to point out differences in the phenotype (e.g. in the wing). We expect minor differences for at least two reasons. 1) The Notch activity appears to be (partially) repressed as opposed to abolished. 2) As we also point out repeatedly, the growth promoting function of α-S might also contribute to the phenotype (our previous publication on this is Ho et al., 2021; DOI: 10.1242/dmm.048132). Furthermore, the fact that o/e NICD and elevated α-S and α-PheRS expression counteract each other in a situation where Notch induces proliferation **and** in a situation where it induces differentiation (and prevents proliferation) makes it very challenging to come up with an alternative hypothesis. As this reviewer acknowledges, the experiments with the Notch reporter, Notch responsive element promoter (NRE)-GFP, then told us that this can indeed happen *in vivo*.

It is important for the authors to show this particularly because there is no biochemical evidence provided that alpha-PheRS competes for factors that interact with the NICD nor evidence of effects on Notch target expression.

➔ We are aware that showing a biochemical interaction is very much desirable. However, doing this *in vivo* is still a challenge, in particular if DNA binding is needed for the interaction (and the α-S fragment contains a DNA binding domain). Tissue culture cells, *in vitro* assays or Y2H would be simpler (and are usually done for similar experiments). But then, one can still argue that these are artificial situations. We truly believe that showing the phenotypic similarities, the genetic interactions between *Notch*^{lof} and elevated α-S and α-PheRS levels in several different situations, and the effects on the Notch target gene promoter are sufficient to claim that they interact and compete, even if the molecular nature of the interaction has not been proven.

Adult midgut: The authors insist that the effects of alpha-PheRS overexpression resemble the loss of Notch in the adult midgut. This is based on an expansion of esg+ cells and a mild increase in Pros+ EE cells. It is important to note that hyperproliferation in the gut can increase number of esg+ cells (e.g., see Biteau et al., 2008). This will also mildly increase Pros+ cells as new Pros+ cells are added to an epithelium already containing Pros+ EEs. The loss of Notch in adult midguts results in an expansion of ISC- like cells that express high levels of Delta (ISC-specific marker), esg, E-Cadherin, Armadillo and Pros (EE marker) (Maeda et al., 2008; Patel et al., 2015; Chen et al., 2018). There are cheap, publicly available antibodies for many of these markers (e.g., Delta, E-Cadherin, Armadillo). These N- ISC-like progenitors are often highly-adherent to each other due to the high E-Cadherin levels. Furthermore, the loss of Notch in adult midgut progenitors results in the formation of clusters of Pros+ EE cells, which is not observed with alpha-PheRS overexpression.

➔ We have been using two of these markers (anti-Pros and esg>*UAS-2XeYFP)* but have not seen the need for using the others as well. We actually have tried a couple more, but it seems that our antibodies did not work (anymore?) and we would have to reorder them and set up the crosses again. This, however, would take too long because the crosses need to be done at 18°C. More importantly, we do not want to claim that the two phenotypes are "identical". We claim that they "resemble" each other. In fact, we see the phenotype caused by elevated α-PheRS levels as a combination of a proliferative activity and a reduced Notch activity. This is also apparent from the subtitle and the conclusion sentence of this result section:
"**The phenotype of elevated α-PheRS levels partially resembles the low Notch phenotype**"
"We conclude that elevated α-PheRS levels in midgut stem cells cause misregulation of gut homeostasis and that this effect is not only the consequence of the proliferative role of α-PheRS but also caused by downregulating Notch signaling and inhibiting the terminal differentiation of the progenitor cells (EBs)." From this it is not clear to us, what the suggested additional work would contribute.

The authors claim that they cannot examine MARCM clones in the midgut to analyze cell number due to cell fate changes. This is not true as MARCM clones can be used to assess cell number within midgut clones despite cell fate changes (see Salle et al., 2017; Lee et al., 2009).

➔ We agreed that MARCM clones can be performed in the midgut to analyze the effect on cell numbers. We would, however, need to construct the required strains because we would have to use the esg^{ts} driver whereas we made the strains for our MARCM analysis in the follicle cells with the tub-Gal4 driver. And these crosses would have to be performed at 18°C. We think that this is not justified because of our previously published results of the analogous MARCM analysis of α-PheRS in follicle cells (Ho et al, 2021; at the single cell level, the higher α-PheRS

levels did not affect cell size, but the total clone size and the cell numbers increased with the higher α-PheRS levels). The follicle cell lineage has the advantage that it does not produce different cell types. Moreover, in the text in the manuscript we again point out the dual effects of α-PheRS overexpression, repressing Notch and stimulating proliferation.

NICD expression in adult midgut progenitors results in rapid differentiation into ECs without affecting existing Pros+ EEs. The authors claim that a loss of EEs upon NICD expression has been described before, but this is not true. The papers cited by the authors (Micchelli and Perrimon, 2006; Zhai et al., 2017) did not examine the effects on Pros+ cells after NICD expression in adult midgut progenitors.

➔ The reviewer is correct about this. We did not read the text properly.
However, the main focus of our experiment was on the interaction between α-PheRS and Notch/NICD for the control of the ISC/EB (and EC) population. We therefore did not follow up on the effect of the NICD on the EE population.

The authors have also not addressed why they utilized Western blot analysis to assess NRE-GFP levels in the midgut. NRE-GFP can be observed in the midgut (see Wang et al., 2020). The authors stated that an internal control compartment is necessary for in vivo analysis, but an experimental midgut can be compared to an independent control midgut not overexpressing alpha-PheRS.

➔Westerns allow more quantitative comparisons than staining experiments with two different organs fixed and stained in different tubes. Having two compartments like in the wing disc (where the internal control is the anterior compartment of the same organ tissue) is so much better than staining two different organs and claiming that the fixation and all of the staining was done exactly the same way. If this is not possible, we prefer to run a Western blot where we have a loading control by probing the blot with an appropriate standard antibody. We do not see the additional value of this experiment and have, therefore, not started it. Should this, however, be an essential addition, we would need some time to set up the required crosses with *Gla80^{ts}*, which have to be done at 18°C.

It is not clear whether the effects of alpha-PheRS overexpression in the larval midgut resemble those of the loss of Notch. The authors show an increase in esg+ cells and a mild increase in Pros+ cells. The authors provide us with a quantification of Pros+ cells in the larval midgut, but do not provide images of this expansion. However, studies that have examined Notch signalling in the larval midgut have not described an expansion of esg+ cells and Pros+ cells. These studies have rather shown that the loss of Notch in the larval midgut results in an increase in pupal endocrine cells within AMP islands and a loss of peripheral cell formation in the larval midguts (Takashima et al., 2011; Mathur et al., 2010). This is something that the authors have not examined.

➔ We had been advised to focus on the adult intestine like most other groups do. We therefore focused on the adult intestine but kept the interesting larval findings we already had (and we never investigated the pupal gut). Because they fit in well with the present story, we decided to add them to this manuscript. The approx. 2.5-fold increase in EEs with α-PheRS^{Cys} we consider a striking increase. We noted that Takashima et al., 2011 did show the increase of pupal EE cells within AMP islets. They also used the same Pros+ marker as we did, and they showed a high increase of Pros^{+} cells when silencing *Notch*. The difference between our study and theirs is that we did not specifically analyze the number of EE cells **within** AMP islets. Because we counted the percentage of Pros^{+} cells per total cell number for the statistics, we come to very similar conclusions.

The authors have shown that the loss of Notch in the larval wing results in a loss of NRE-GFP and a notched wing. In contrast, the alpha-PheRS overexpression, which results in a loss of NRE-GFP, does not cause a notched wing. These differences have still not been reconciled by the authors. The authors suggest that compensatory proliferation helps alpha-PheRS overexpressing wings to reach proper organ size, but the authors have not provided this data.

➔ Because the effects of elevated α-PheRS levels on the NRE activity is so strong in the wing discs, it appeared unlikely that this is simply a mild phenotype. Given that we had previously identified the proliferative activity of α-PheRS$^{(Cys)}$ (Ho et al., 2021) it seemed more likely that this activity manages to compensate for loss of the Notch activity that would otherwise cause the notched wing. The idea of compensatory proliferation is consistent with the observed increase in PH3^{+} cells (mitotic index, discussed in the discussion of the manuscript) we observed in the wing discs.
(We do not have proof for this - and we do not present it as a result either - but in two independent assays (IP and proximity labeling) we have obtained (yet unconfirmed) results that show interactions between α-S/PheRS and components of the Hippo organ size control mechanism).

The authors claim that alpha-PheRS overexpression blocks Notch signalling in larval neuroblasts, which results in the loss of type 1 and type 2 neuroblasts. However, Notch is not required for in type 1 neuroblast maintenance (Haenfler et al., 2012), but it is required for type II neuroblast maintenance (Haenfler et al., 2012; Xiao et al., 2012; Li et al., 2016). To show that the loss of type 2 neuroblasts is due to inhibiting Notch, the authors need to show that they are lost by alpha-PheRS overexpression due to molecular changes that occur due to the loss of Notch signalling in type 2 neuroblasts (e.g. erm expression, Li et al., 2016). From the data provided, it's not clear if their loss is due to affecting Notch or by affecting another process. Are these type 2 neuroblasts overexpressing alpha-PheRS lost due to cell death or by differentiation or by transformation to type 1 neuroblasts?

➔ This was indeed not properly described by us in the revised manuscript because we had to add another part about the type I neuroblasts, and we did not adjust phrasing properly in some of the older neuroblast sections. This was the case in the abstract and in the introduction to the experiment (p12). We had now specified where we refer to type II neuroblasts only.

In the previous response to the reviewer's request for a deeper analysis of how this phenotype comes about, we had stated the following (which has not changed since. "With this experiment we only wanted to test whether α-PheRS indeed acts on Notch signaling or whether it affects primarily proliferation and not Notch signaling. This system allowed us to do this because in (type II) NBs, *N* signaling has the opposite effect on proliferation vs differentiation. Figuring out the details of this pathway could easily be an independent project. We added the images for loss of Notch and the effect of α-$PheRS^{Cys}$ and NICD on type 1 neuroblasts as requested." Following the latter request may have been a mistake in hindsight. It was never our intension to elucidate the molecular mechanism of the effects on all NBs. The reason for this experiment was only to test the hypothesis that α-$PheRS^{(Cys)}$ levels counteract Notch signaling in a situation where Notch promotes proliferation and not differentiation (as in the gut).

Lastly, their new staining for alpha-PheRS in Fig. 1 does not support their claim that alpha-PheRS is enriched in progenitors. It seems to be expressed in all the cell types and seems enriched in some Pros+ cells, but not all (see Fig. 1E"). Thus, the authors should reconsider their statement about alpha-PheRS being enriched in stem cells/progenitors.

➔ The reviewer is correct, in the esg^{ts}>eYFP labeled adult gut we present (in Fig 1E-F), the YFP-labeled cells show only a slightly higher signal intensity for α-PheRS. Because these YFP+ cells have only very little cytoplasm, this signal gets almost lost and there is less overall signal in these cells than in the EEs. We have now rephrased this part in the result section in a more conservative way.

Reviewer #2: The authors now have strengthened an antagonizing function of alpha-PheRS on the Notch signaling pathway by additional experiments. Additional control experiments were also performed. Unfortunately, despite several approaches, a direct interaction of alpha-PheRS with Su(H) at DNA could not be shown. However, the proposed mechanism was now more cautiously considered as "possible" in several places in the manuscript by the authors. The title of the manuscript has also been changed accordingly. I have no additional criticisms or objections.

Reviewer #3: In this revised version the authors addressed carefully the majority of my comments and suggestions. Last major concern is still the technical problem of the image quality commented on before. The authors state that this was improved, but the downloadable PDF is still having the problem of poor image resolution which leaves me unable (again) to see proper images and/or read Y-Axis (Fig.2+6). So, I cannot judge this, but this needs to be sorted out between the editor and authors before publishing. Overall, when these issues are fixed I recommend this manuscript for publication and congratulate the authors for this nice work.
