# Supplementary material for: α-Phenylalanyl tRNA synthetase competes with Notch signaling through its N-terminal domain
Source: PLoS Genet. 2022 Apr 29;18(4):e1010185. doi: 10.1371/journal.pgen.1010185 (PMC9094542; doi:10.1371/journal.pgen.1010185)
Supplement: Attachment — Submitted filename: PGENETICS-D-21-01190_Review.docx [file pgen.1010185.s005.docx]

The authors identify the alpha subunit of the cytoplasmic Phenylalanyl tRNA synthetase (α-PheRS) as a novel antagonist of the Notch signaling pathway in drosophila.

Using defined fly strains, to express or knock-down, the components/targets in a tissue and/or temperature dependent manner, they show convincingly repression of Notch phenotypes and downregulation of a Notch responsive reporter by α-PheRS in three different settings: midgut, neuroblasts (NBs) and wing formation.

They also show that the N-terminal part of the protein (alpha-S) is sufficient for repression. In addition, a tryptophan residue within alpha-S seems to be essential for repression of the Notch signaling pathway. Mechanistically, they propose a model of action (Notch repression) in which α-PheRS (alpha-S) competes for NICD/Su(H) interaction by binding to the BTD of Su(H) as the RAM domain of NICD does. This mechanism was shown to be realized by RBPJ-corepressor action in vertebrates.

The MS is well written and the experiments are sound and conclusive. I have to congratulate the authors for this peace of work. If true, they discovered a novel mechanism for Notch modulation (here repression) in the fly, not known so far.

However, showing the displacement mechanism to repress Notch needs to be improved. Therefore, I think some points have to be clarified by experiments and/or discussion prior to publication:

1. Although the authors mention a compensation of alpha-PheRS(W112A) for the wt form regarding the Phe-t-RNA loading function, expression of alpha-S(W112A) and alpha-PheRS(W112A) should be shown by WB (anti-Myc).

2. Is alpha-PheRS (alphaS) located in the nucleus and are the mutated forms still located in the nucleus? (microscopy using higher magnification should be performed).

3. To strengthen the proposed model of action of alpha-PheRS repressing Notch signaling (which is weak so far), Co-IPs with alpha-PheRS(wt), alpha-S(wt), together with Su(H) are necessary. alpha-PheRS (W112A) and/or alpha-S(W112A) should be used as control.

If the N-terminal part of alpha-S (especially the residues around the tryptophan (HGWIL) is conserved also in vertebrates (alignment as additional figure), these experiments could also be performed using vertebrate proteins.

4. Does the proposed displacement mechanism occur on Notch target genes?

A ChIP analysis would be helpful but might also be a difficult task in drosophila tissues.
